# Association of Fine-Needle Aspiration of Thyroid Nodules With Final Histopathology in Diagnosing Thyroid Malignancy: A Single Institute Retrospective Study

**DOI:** 10.7759/cureus.31733

**Published:** 2022-11-21

**Authors:** Ali S Alshahrani, Alhassan G Algazlan, Montasir Junaid, Abdulrahman A Aldosari, Khaled A Amer, Musleh H Mubarki, Salmah M Alharbi, Ali M Al-Qannass

**Affiliations:** 1 Otolaryngology-Head and Neck Surgery, Armed Forces Hospital Southern Region, Khamis Mushait, SAU; 2 Otolaryngology-Head and Neck Surgery, Aseer Central Hospital, Abha, SAU; 3 College of Medicine, King Khalid University, Abha, SAU

**Keywords:** hsitopathology, fine needle aspiration cytology (fnac), correlation, concordance, histopathology, fnac, lesion, thyroid nodule

## Abstract

Background

Fine needle aspiration cytology (FNAC) is the gold standard for detecting thyroid nodules. It is a cost-efficient approach that affords prompt and accurate evaluation. It is crucial in deciding to treat patients with suspected malignancy of thyroid nodules that might have thyroid surgery. According to findings in cytology, patients may be observed when the cytology is benign, and surgery can be performed if the cytology is malignant, which leads to a reduction in the incidence of unneeded surgery.

Aim

The current study aims to assess the concordance between FNAC of thyroid nodules with final histopathology and identify the different types of detected thyroid lesions.

Methodology

A retrospective record-based study reviewed the medical files of all patients presenting to the Armed Forces Hospital, Southern region, with suspected thyroid nodules from April 2018 to January 2020. Data were extracted using pre-structured data extraction sheet to avoid inconsistency. Data extracted included patients' demographic data, swelling laterality, size, ultrasound, and histopathological findings.

Results

The baseline characteristics of studied samples in the present study: Forty-seven samples had a mean age of 44.27 (SD=±13.5) years, and 85.1% were female gender. The study showed that 12.5% of benign samples were lymphocytic in histopathology, 25% suspicious for follicular neoplasm samples were benign multi-nodular goiter in histopathology, and all 100% of samples suspicious for malignancy were malignant in final histopathology.

Conclusions

The current study showed that the malignancy rate of the examined nodules was not uncommon in FNAC and histopathology. Where papillary carcinoma was the most detected malignancy, the diagnosis of malignancy using FNAC is a cost-efficient approach that affords prompt and accurate evaluation. Once diagnosed, these cases should be subjected to surgery.

## Introduction

A solitary thyroid nodule is defined clinically as a localized thyroid enlargement with an apparently normal remaining gland. A solitary thyroid nodule is a common thyroid condition. The major objective of examining these nodules is to identify those with malignant potential [1,2]. Thyroid nodules estimated incidence was up to 50% of subjects. Most nodules are benign, but about 3%-7% of cases are found to be malignant in the United States in 2005 [3]. To diagnose thyroid nodules, physicians can use a diagnostic test, such as ultrasonography, thyroid scintigraphy, and fine-needle aspiration cytology (FNAC) [4]. FNAC is an efficient test that is a standard test for thyroid nodule diagnosis [5].

Physicians still face challenges regarding evaluating thyroid nodules among patients with thyroid disease. When performed in the outpatient setting, fine-needle aspiration cytology is a prompt, simple, and reliable diagnostic tool with high patient compliance. A typical specimen from the nodule, with the results interpreted by a skillful cytologist, is a significant factor in an adequate test [6]. It is often the first step in diagnosing thyroid nodules [7].

Ultrasound-guided fine-needle aspiration biopsy (UGFNAB) enables continuous imaging of needle insertion and sample collection, allowing for the needle's location within the lesion to be confirmed with confidence and ease. Small solid and cystic suspicious nodules can be discovered and biopsied using a needle that can be directed to the solid area [8,9].

This study aimed to compare the FNAC results of thyroid nodules with the final histopathology results and to include the different types of thyroid lesions that may have been found.

## Materials and methods

A retrospective record-based study was conducted, which reviewed the medical files of all patients presenting to the Armed Forces Hospital, Southern region, Saudi Arabia, with suspected thyroid nodules from January 2018 to January 2020. Any cases with neck swelling other than thyroid nodules were excluded. Also, medical files with incomplete/missing relevant data were excluded. There was no contact or risk to patients as this is an audit of the last two years, and all patients have been already operated on/treated. Data were extracted using pre-structured data extraction sheet to avoid inconsistency. Data extracted included patients' demographic data, swelling laterality, size, FNAC results, and histopathological findings.

Statistical analysis

Data have been saved and analyzed with the use of IBM Corp. Released 2015. IBM SPSS Statistics for Windows, Version 23.0. Armonk, NY: IBM Corp. Counts with percentages were reported for baseline characteristics, ultrasound findings, FNAC, histopathology findings, and other studied parameters of studied samples, mean with standard deviation or median with the range given for quantitative parameters. Association of ultrasound findings, FNAC, and histopathology were tested using Pearson Chi-Square at a 5% significance level. A pie diagram was also used to present data graphically.

## Results

Table 1 reports the baseline characteristics of the studied samples; in the present study, 47 samples had a mean age of 44.27 (SD=±13.5) years, 85.1% were females, the majority, 85.1% with multiple nodules, the median size of the nodule on Ultrasound was 3 cm with range 0.6 to 14 cm on average.

**Table 1:** Descriptive Statistics on Gender, Neck Swelling, and Ultrasound Findings (n=47)

Variables		n	%
Gender	Female	40	85.1
	Male	7	14.9
Age (years)	Mean ±SD	44.27	±13.5
Neck swelling	Single Nodule	7	14.9
	Multiple Nodule	40	85.1
Ultrasound findings "TI-RADS Score"	TR1	10	21.3
	TR2	12	25.5
	TR3	6	12.8
	TR4	18	38.3
	TR5	1	2.1
Size of the nodule in cm on ultrasound or the largest nodule in multi-nodular goiter	Median (Range)	3.0 cm	(0.6 - 14 cm)

**Figure 1:**
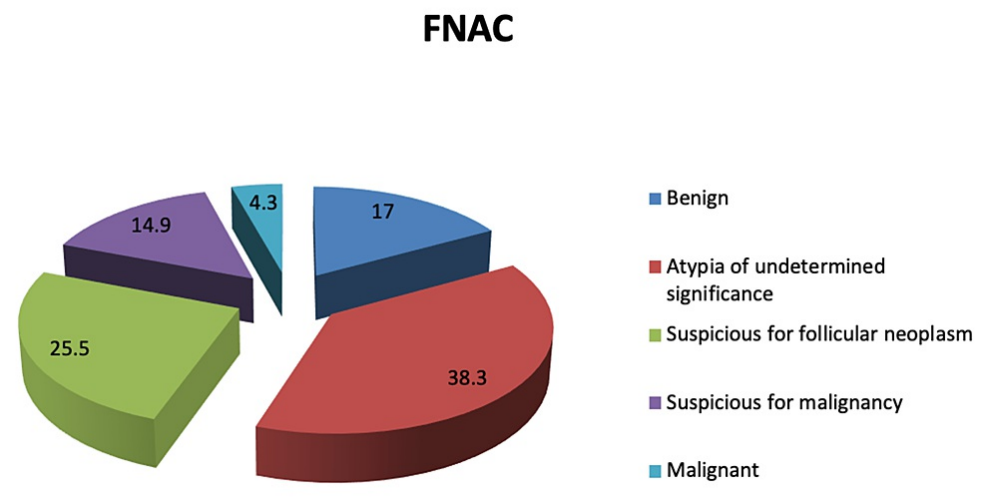
FNAC of Thyroid Nodules FNAC: Fine-needle aspiration cytology

Table 2 reports according to FNAC, 38.3% were atypia of undetermined significance, 14.9% were suspicious malignancy, and 4.3% were malignant. Figure 1 also represents the outcomes of FNAC.

**Table 2:** Thyroid FNAC Results (Bethesda system) FNAC: Thyroid fine-needle aspiration cytology

FNAC	n	%
Benign	8	17.0
Atypia of undetermined significance	18	38.3
Suspicious for follicular neoplasm	12	25.5
Suspicious for malignancy	7	14.9
Malignant	2	4.3

Table 3 gives the outcomes of histopathology. 31.9% of samples were found with benign multinodular goiter, and 46.8% were malignant.

**Table 3:** Final Histopathology Result NIFTP: Non-invasive follicular thyroid neoplasm with papillary-like nuclear features

Histopathology result	n	%
Lymphocytic thyroiditis	2	4.3
Benign multinodular goiter	15	31.9
Benign solitary nodule	4	8.5
Pre-malignant: NIFTP	4	8.5
Malignant	22	46.8

Table 4 reports the association between FNAC and outcomes based on the histopathological diagnosis. Results showed 12.5% of benign samples were lymphocytic in histopathology, 25% suspicious for follicular neoplasm samples were benign multinodular goiter in histopathology, and all 100% samples of suspicious for malignancy were malignant in histopathology. Pearson Chi-Square test did not give any significant association between FNAC and histopathology p=0.107.

**Table 4:** Association of Final Histopathology With FNAC FNAC: Thyroid fine-needle aspiration cytology, NIFTP: Non-invasive follicular thyroid neoplasm with papillary-like nuclear features, P=0.107 using Pearson Chi-Square test

Final histopathology	FNAC results (Bethesda system)									
	Benign		Atypia of undetermined significance		Suspicious for follicular neoplasm		Suspicious for malignancy		Malignant	
	n	%	n	%	n	%	n	%	n	%
Lymphocytic thyroiditis	1	12.5	0	0.0	1	8.3	0	0.0	0	0.0
Benign multinodular goiter	3	37.5	9	50.0	3	25.0	0	0.0	0	0.0
Benign solitary nodule	0	0.0	2	11.1	2	16.7	0	0.0	0	0.0
Pre-malignant : NIFTP	0	0.0	1	5.6	3	25.0	0	0.0	0	0.0
Malignant	4	50.0	6	33.3	3	25.0	7	100.0	2	100.0

## Discussion

Fine needle aspiration cytology (FNAC) is the foremost approach for evaluating thyroid nodules. FNAC is a quick, consistent, minimally invasive, and cost-efficient approach. A primary advantage of FNAC is that it can be completed as an outpatient method [10]. The main objective is to differentiate benign from neoplastic or malignant thyroid nodules. Literature showed that FNAC has led to a considerable decrease in the frequency of surgeries among patients with thyroid nodules and a higher number of operated malignant lesions [11]. Recently, this technique has been used in clinical practice worldwide [12,13]. The limitation of FNAC includes false-negative and false-positive results. Physicians should know about these possible disadvantages and drawbacks of FNA interpretation, such as specimen inadequacy, sampling techniques, and others [14].

The current study assessed the FNABC of thyroid nodule correlation with final histopathology. The study results showed that more than one-third of the thyroid nodules were Atypia of undetermined significance, 14.9% were suspicious malignancies, and only 4.3% were malignant based on FNAC.

Histopathology showed that malignant nodules were dominant (46.8%), followed by benign multi-nodular goiter (about one-third of the nodules). Papillary carcinoma was the most diagnosed malignancy among examined nodules. As for the association between FNAC and histopathology findings, the current study showed that 12.5% of benign samples were lymphocytic in histopathology and 25% suspicious for follicular neoplasm samples were benign multi-nodular goiter in histopathology, and all samples suspicious for malignancy were malignant in histopathology. This result means high concordance between FNAC and histopathological evaluation of the thyroid nodules among the current study patients, especially malignant nodules. As per Chirag P et al. FNAC was assessed to have a diagnostic accuracy of 94.5%, with a sensitivity of 84.2% and a specificity of 97.2%, respectively. The positive predictive value was 88.3%, whereas the negative predictive value was 96%. Papillary thyroid carcinoma was the most common malignancy identified [15]. Similar results were reported by other research where Bouvet et al. estimated sensitivity, specificity, and accuracy of FNAC of 93.5%, 75%, and 79.6%, respectively [16], and Kessler et al. estimated values were 79%, 98.5%, and 87%, respectively [17]. Other studies revealed that the incidence of false-negative for FNAC ranged from 1% to 30%, with a low false-positive rate (13.3%) for neoplastic lesions, but none were malignant [18,19]. Machała E et al. reported that FNAC has a sensitivity of 60.28% and a specificity of 98.05%. The rate of false positives was 1.95%, and the rate of false negatives was 39.72%. The positive predictive value was 90.1%, and the negative predictive value was 89.35%. The accuracy of FNA in distinguishing benign from malignant thyroid lesions was 89.46%, which is high, indicating that FNAC has a superior performance in evaluating malignant variations [20]. In our study, it was also found that all malignant nodules found by FNAC were also found to be malignant by histopathology. This result fits with similar findings from other studies [17,20].

Some studies concluded that assessing the valid rate of false-negative results is difficult as only about 10% of patients with benign cytologic findings have undergone surgery [21,22]. Also, they reported the limited ability of FNAC to precisely identify follicular pattern lesions, cystic papillary thyroid carcinoma (PTC), and papillary microcarcinoma [23]. This may be due to the incidence of diagnostic features of papillary cancer, even in benign conditions such as adenomatous goiter, thyroiditis, nodular goiter, and follicular neoplasm [21,24].

## Conclusions

The current study showed that the malignancy rate of the examined nodules was not uncommon in FNAC and histopathology, where papillary carcinoma was the most detected malignancy. Regarding concordance between FNAC and histopathological assessment of the thyroid nodules, the study showed a satisfactory agreement rate between FNAC and histopathological findings, especially for malignant features. This study shows that diagnosing malignancy using FNAC is easy, cost-efficient, and demonstrates accuracy in the case of thyroid malignancy. Once diagnosed, these cases should be subjected to surgery.
